# Using informatics to advance translational science: Environmental scan of adaptive capacity and preparedness of Clinical and Translational Science Award Program hubs

**DOI:** 10.1017/cts.2022.402

**Published:** 2022-05-16

**Authors:** Bart Ragon, Boris B. Volkov, Chris Pulley, Kristi Holmes

**Affiliations:** 1 Integrated Translational Health Research Institute of Virginia, Charlottesville, VA, USA; 2 University of Virginia, Charlottesville, VA, USA; 3 University of Minnesota Clinical and Translational Science Institute, Minneapolis, MN, USA; 4 Institute for Health Informatics and Division of Epidemiology and Community Health, University of Minnesota, Minneapolis, MN, USA; 5 Northwestern University Clinical and Translational Sciences Institute, Chicago, IL, USA; 6 Department of Preventive Medicine, Northwestern University Feinberg School of Medicine, Chicago, IL, USA

**Keywords:** Informatics, Clinical and Translational Science Award Program, translational science, adaptive capacity, emergency preparedness, environmental scan

## Abstract

As the USA and the rest of the world raced to fight the COVID-19 pandemic, years of investments from the National Center for Advancing Translational Sciences allowed for informatics services and resources at CTSA hubs to play a significant role in addressing the crisis. CTSA hubs partnered with local and regional partners to collect data on the pandemic, provide access to relevant patient data, and produce data dashboards to support decision-making. Coordinated efforts, like the National COVID Cohort Collaborative (N3C), helped to aggregate and harmonize clinical data nationwide. Even with significant informatics investments, some CTSA hubs felt unprepared in their ability to respond to the fast-moving public health crisis. Many hubs were forced to quickly evolve to meet local needs. Informatics teams expanded critical support at their institutions which included an engagement platform for clinical research, COVID-19 awareness and education activities in the community, and COVID-19 data dashboards. Continued investments in informatics resources will aid in ensuring that tools, resources, practices, and policies are aligned to meet local and national public health needs.

## Introduction and Background

Informatics has long played a significant role in the Clinical and Translational Science Award (CTSA) Program, illustrated through the Program’s 2018 Funding Opportunity Announcement (FOA). Biomedical informatics has been described as “a critical Program focus for enabling and advancing translational research, which is increasingly data intensive and requires collaboration across many communities, including healthcare, research, and public health” [1]. CTSA hubs have been guided to integrate ever-increasing amounts and types of data to be used to generate knowledge; to use informatics across the entire spectrum of the hubs’ translational research activities, supporting pre-clinical and clinical research, community engagement, education, and training. Likewise, the integration of data from diverse sources (e.g., clinical and research databases/datasets, sensors, mobile technology, social media, patient-based and public data) requires hubs to provide research teams with user-friendly data management systems bolstered by proper training and assistance for their use, which is a persistent and critical challenge. The role of informatics in supporting the goals of advancing clinical and translational science is a significant aspect of the most recent 2021 FOA, with “well-integrated and developed informatics and digital health capabilities” highlighted as one of the “essential characteristics of successful CTSA Hubs” [2]. Informatics will continue to play a major role in the ongoing evolution and continuous quality improvement of the CTSA Program.

Informatics capabilities and a commitment to open science principles across all aspects of the CTSA hub are critical to a successful clinical and translational science environment that can translate knowledge into practice and improve health. The capability to share and implement resources across CTSA hubs, when appropriate, offers opportunities to accelerate scientific discovery as well as improve the efficiency, quality, and impact of translational research [2].

The CTSA Program’s history of developing informatics infrastructure allowed many organizations to quickly respond to the COVID-19 pandemic. Informatics has directly supported the advancement of “patient care, clinical decision support, training researchers and practitioners, as well as public health surveillance and clinical research to levels that could not have been accomplished without the years of ground-laying work by the CTSAs” [3]. In 2021, the CTSA Steering Committee approved the Adaptive Capacity and Preparedness (AC&P) Working Group to identify, curate, analyze, and share examples of practices, challenges, and lessons learned related to how CTSA hubs responded and adapted to a public health crisis [4]. This paper is part of the Working Group’s Environmental Scan of Adaptive Capacity and Preparedness of CTSA Hubs, which was not intended to evaluate, test, generalize, quantify, or validate any hypotheses, approaches, or interventions [4], but rather to illuminate relevant examples from CTSA hubs to help them build and augment their adaptive capacity to effectively respond to future events. Jones *et al.* [5], in their presentation of the Local Adaptive Capacity (LAC) Framework, describe how understanding adaptive capacity at a local level can lead to a more comprehensive understanding at a national level. To guide our exploration of adaptive capacity and preparedness, we will apply the following key characteristics of the LAC framework: asset base (key assets that allow hubs to respond to evolving circumstances); institutions and entitlements (an appropriate and evolving institutional environment that allows fair access to key assets and capitals); knowledge, information, and learning (the ability to collect, analyze and disseminate knowledge and information to learn in support of adaptation activities); innovation (an enabling environment to foster innovation, experimentation, and the ability to explore pragmatic solutions and opportunities); and flexible forward-looking decision-making and governance (the ability to anticipate and respond to changes with regards to its decision-making, governance, and operational structures). This approach can serve as a platform to monitor progress, identify needs, and develop resources that enhance the system’s ability to adapt to change.

## Asset Base

According to CARE International, “…resilience is increased if the capacities and assets to deal with various shocks, stresses and uncertainties are built and supported” [6]. For years, CTSAs have been building informatics infrastructure to support clinical and translational research. The complex relationship between assets and adaptive capacity ensures that access to appropriate resources can limit or enhance an organization’s ability to respond to a crisis. Traditional frameworks have an asset-oriented approach, which can be useful for understanding the resources within the system but fail to illustrate the role of process and its ability to support adaptive capacity. The Local Adaptive Capacity framework helps to illustrate the connection of the asset base to the rest of the system at a local level [5].

To support the CTSA Program goals of making headway in clinical and translational science and improving the quality and efficiency of clinical research, CTSA institutions are now required to build and utilize a solid base of assets (infrastructure, expertise, and capabilities) in the areas of: Health Informatics, applying informatics research and practice throughout clinical and health domains; Clinical Research Informatics, supporting the discovery and management of health and disease information from and for clinical trials and secondary use of clinical data; and Translational Bioinformatics, generating storage, analytic, and interpretive methods to translate immense biomedical data into “proactive, predictive, preventive, and participatory health” [2]. Along with the above areas, CTSA institutions are also encouraged to build innovations on processes and programs that advance translation through collaborative structures, project management, and incentivization for team science.

Prior to the pandemic, researchers’ asset base was defined by their ability to access organizational electronic health records or consortia of local and regional networks. In retrospect, that standard was narrow, myopic, and entirely inadequate to address a major public health crisis (CTSA informatics expert/stakeholder, email communication, January 24, 2022). Haendel *et al.* [7] note how to access the consortia networks such as Accrual to Clinical Trials (ACT) Network, National Patient-Centered Clinical Research Network (PCORnet), and Consortium for Characterization of COVID-19 by EHR (4CE) supported querying structured data, but limited researchers due to their distributed nature; the authors argue for a centralized resource to enable rapid integration. The National COVID Cohort Collaborative (N3C), originating from the CTSA Center for Data to Health (CD2H), is designed to aggregate and harmonize COVID-19 clinical data across organizations through a partnership with CTSA program hubs.

One example of a practical, adaptable informatics asset is the Profiles Research Networking Software supported by Harvard Catalyst – an open source database of profiles of thousands of faculty from Harvard Schools of Dental Medicine, Medicine, and Public Health, used pre-pandemic by many other organizations and investigators as a tool to search beyond an individual hospital’s borders. During the pandemic, Profiles software was selected to facilitate the work of an international 4CE consortium that uses EHR data to understand COVID-19, represented by over 100 investigators across different specialties, including informatics, statistics, and clinical medicine. Profiles software was adapted to address the need for sharing clinical research information about COVID by processing information on thousands of PubMed articles to automatically generate profiles for all investigators involved [8].

The *Journal of Clinical and Translational Science*’s (JCTS) special issue on COVID-19 sought to capture best practices from the pandemic [9]. As part of that special issue, Coller *et al.* [10] developed a checklist for CTSA program hubs to consider when responding to future similar public health emergencies. The checklist’s four major focus areas include Institutional Administration, Individual Departments and Units, Human Research Protection Plan and IRB, and CTSA Hub (Institutional Translational Center or Institute). Peppered throughout the checklist are considerations for policy creation and alignment, communication strategies, bi-directional partnerships, customized data dashboards, ensuring access to high-quality data. Hubs may wish to consider concepts from the checklist when reviewing their asset base to bolster their ability to respond to future events. Specific to informatics support are recommendations for adopting novel informatics platforms, hiring individuals with required informatics skills while considering work from home policies, and strengthening security, privacy, and technical capability of informatics platforms.

## Institutions and Entitlements

This LAC framework domain is interconnected with the Asset Base domain and deals with the availability of an appropriate and evolving institutional environment that allows fair access and entitlement to key assets (including precious knowledge and information). Indeed, a successful clinical and translational science environment that can translate knowledge into practice and improve health is also determined by the presence of strong informatics capabilities and a commitment to open science principles across CTSAs and all their aspects. Co-creating, sharing, and implementing resources across CTSA hubs catalyze scientific discovery and opportunities to enhance the efficiency, quality, and outcomes of translational and clinical research [2].

**Fig. 1.**
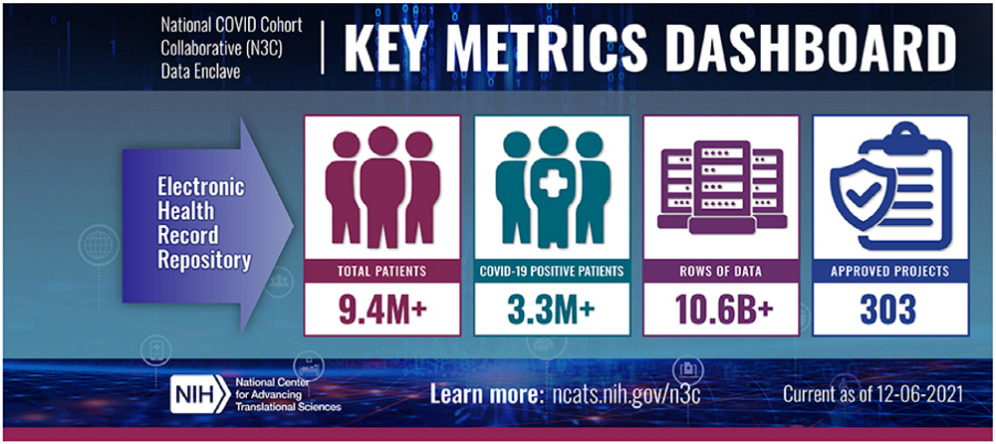
N3C key metrics dashboard [12].

The collaborative, creative environments and resources of NCATS, CTSA hubs, the National Center for Data to Health (CD2H), and NIGMS-supported Institutional Development Award Networks for Clinical and Translational Research (IDeA-CTR) have been coalesced through a N3C partnership to contribute and utilize COVID-19 clinical data to address pandemic research questions through four goals: (1) create a robust data pipeline to harmonize EHR data into a common data model; (2) provide easy access for the clinical and research community to a wealth of COVID-19 clinical data; (3) establish a resource for the next 5 years to understand long-term health impact of COVID-19; and (4) create a cutting-edge analytics platform to enable COVID-19-related novel analyses. The volume of the N3C robust and securely shared data has been growing (Fig. 1), and so is the partnership’s impact on clinical research and practice, translated into public health benefits. The N3C partnership suggests that a collaborative analytics approach could be adopted and adapted for addressing other diseases, translating to our collective research capacity and preparedness for future public health emergencies. This was demonstrated by N3C Domain Teams, which enabled researchers with shared interests to efficiently analyze data and collaborate within the N3C Data Enclave’s team science environment. Clinical Domain Teams include multidisciplinary, clinical and subject matter experts, statisticians, informaticists, and machine learning specialists that collaborate on clinical questions related to COVID-19’s impact on health conditions, whereas Cross-Cutting Domain Teams’ foci are distributed across multiple domains [11].

A major theme detected by Bookman *et al*. was an increase in the speed with which decisions were made [3]. The needs caused by the pandemic allowed for risk tolerance to be recalibrated, for example, in telemedicine, allowing for condensed decision timelines. Results from the CTSA informatics survey illustrate increased use in research data infrastructure, increased data warehouse refreshes, rapid cohort identification of COVID-19 patients for research, and increased utilization of informatics resources due to COVID-19. The COVID-19 pandemic presented a challenge for policymakers who were required to react to an emerging threat and make decisions, even though the extent of the threat was unknown [13]. Quick actions from CTSA hubs certainly assisted in response to the pandemic but the impact on patient outcomes or significant discoveries about the pathophysiology and therapeutics for the disease is yet to be determined.

A different survey administered by Croker *et al.* [14] revealed a major shift in biorepository models and how CTSA hubs were able to quickly respond to institutional needs at the onset of the pandemic. Biorepository resources depend on sophisticated tracking systems (e.g., OnCore biospecimen management (BSM), OpenSpecimen, Velos eSample, or customized REDCap models). The specimens’ linkage to clinical data is paramount, and sometimes informatics platforms (such as i2b2) incorporate and allow digital access to the annotated samples, supporting specimen discovery derived from clinical criteria. To support rigorous, reproducible study designs and to further refine phenotype, biorepositories, and informatics teams collaborate on curating concepts, like comorbid conditions and other variables that may not reflect unique variables.

Some experts [3] have observed that despite the increased use of informatics resources, academic medical centers were inadequately prepared for the COVID-19 pandemic. According to the authors, “The pandemic has been a stress test to the system, and it’s useful to consider where strengths and weaknesses were identified so that our institutions are better prepared to respond to the next challenges of the current pandemic and to manage better when the next one strikes.” The maturity of an organization can impact the entitlements available within the ecosystem. Assessing the maturity model of an organization can serve a wide range of functions from identifying system gaps to opportunities for process improvement [15].

Electronic data warehouses for research (EDW4R) are examples of informatics resources important for researchers to access in an efficient, timely manner so they can respond to a crisis with data needed for decision-making. To that end, Campion Jr. *et al*. [16] interviewed CTSA hubs to understand how these hubs defined their EDW4R to support researchers doing clinical and translational science. They identified 12 themes to create a shared understanding about how EDW4R operates across hubs: organization and data; oversight and governance; data access request process; data access modalities; data access for users with different skill sets; engagement, communication, and literacy; service management coordinated with enterprise information technology; service management coordinated within a CTSA hub; service management coordinated between informatics and biostatistics; funding approaches; performance metrics; and future trends and current technology challenges [16]. These 12 themes also relate to the other LAC domains described in this paper.

McClay and colleagues [17] note that electronic health record (EHR) systems are an important informatics resource with the potential to improve healthcare outcomes. Citing issues with research investigators accessing EHR data, they proposed a model of dedicated resources to improve access to EHR data [17]. Resources in the CTR Data Analytics Hub focus on team science and promoting a range of projects from local phenotypes to national studies. Interview responses from investigators supported participation in research networks that could pool these resources across health systems, engage scientists in rural areas, and continue COVID-19 research.

## Knowledge, Information, and Learning

Successful adaptation requires an understanding of the need to adapt, knowledge of available options, and the capacity to implement appropriate measures. Knowledge on the impacts of a significant challenge is a key factor in developing an informed policy response [18]. Learning, as an attribute closely linked to the livelihood of a system, is related to the ability of the system to create new knowledge and strengthen capacity [19]. Informatics is uniquely positioned to contribute to this, as it encompasses all the necessary capabilities to bring together the data, tools/infrastructure, and analytic expertise necessary to study translational science effectiveness and efficiency, as well as to mine it to discover process vulnerabilities, improvements, and create generalizable knowledge to be shared across other CTSA sites. This is evident in the latest Clinical and Translational Science Award [2], where informatics is seen as an innovative solution with the goal of improving health outcomes. In the announcement, health informatics programs, committed to open science principles, are poised to increase the quality and efficiency of clinical research and viewed as a critical resource in translating knowledge into practice [1]. As such, the LAC framework suggests that communities and, by extension, organizations like CTSA hubs share both informal and formal knowledge sources to maximize knowledge generation [5].

The integrated efforts of CTSA hubs and their health care and research partners should lead to dissemination of newly found or optimized knowledge and information (not only about specific diseases or areas of public interest, but also about processes and best practices) to biomedical and translational scientists, clinicians, patients, and other stakeholders. In this pursuit, embracing and cultivating a culture of Open Science and Data Sharing that promotes (Findable, Accessible, Interoperable, Reusable) principles is required for all collaborating organizations [2,20,21]

As COVID-19 emerged, CTSA program hubs rallied to support their organizations and the communities with which they collaborate. Locally, many institutions and CTSA hubs experienced an increased demand for informatics resources. Nationally, the Center for Leading Innovation & Collaboration (CLIC) pivoted to share best practices and lessons learned. The CLIC formed the CTSA Program Response to COVID-19 Discussion Forum, a space for CTSA researchers to discuss organizational efforts connected to COVID-19, including issues related to the use of cutting-edge informatics. The CLIC also created a COVID-19 tag to make resources related to the pandemic easier to locate on COVID-19 information within their website [22].

Our analysis of the JCTS COVID-19 Special Edition Survey [23] revealed common themes from responses about research process, impact, and innovations. Some hubs created de-identified data sets and new data repositories that researchers could use to analyze COVID-19 and non-COVID-19 related data. Researchers used a variety of strategies and tools that allowed them to prioritize COVID-19 research. For example, hubs utilized a dedicated task force/committee and approval process for COVID-19 research. To provide structured, secure access to all COVID-19-related data available, hubs also created new tools (e.g., COVID-19 data marts, dashboards) or used current ones (medical records) to assess COVID-19 and non-COVID-19 research.

One of the examples of a vital role of informatics in learning and informing research during an emergency is a collaboration of the University of North Carolina COVID Recovery Clinic, UNC TraCS, and the Department of Infectious Diseases to resolve the challenging research problem of collecting consistent data on long-COVID patients after they have left the hospital. Utilizing their collective expertise in informatics and following cohorts over long periods of time, these partners created a database that collects patient data for use in clinical research, including a patient’s experience with COVID-19, symptoms during and after infection, pre-existing medical conditions, and demographic information [24]. Another Health Status registry, developed by the integrated Translational Health Research Institute of Virginia (iTHRIV) [25], collects data on healthy and sick people living in Virginia to support COVID-19-related research. The registry’s data helps fill a gap in the knowledge about pandemic and enables communities to make informed decision making for better response and adaptation to the COVID-19 health crisis.

According to Verma *et al.*, one of the biggest challenges in managing patients with COVID-19 is that their ability to respond to treatment is dependent on a number of variables including “age, sex, occupation, community characteristics, social determinants of health, comorbidities, medications, and host genetics” [22]. Thus, it is vital to have or develop agreed-upon data standards that can be captured passively or with minimal interference in care and shared across multiple studies.

The CTSA Program Steering Committee partnered with the JCTS to capture best practices from CTSA hubs on re-engineering the clinical research enterprises while they were responding to the pandemic, so that the information could be shared and to assist with preparing for the future [9]. Within that JCTS issue are several articles discussing COVID-19 research, its impact on clinical research during the pandemic, and the role of CTSA program hubs in supporting research and informatics at academic medical centers. The CLIC portal also dedicated resources supporting CTSA program hubs, including a webinar on “I2I: Research in the Time of COVID-19” [26]. As of November 2021, 530 resources on the CLIC portal were tagged “Covid-19,” and search yielded 82 resources related to COVID-19 and Informatics.

## Innovation

The pandemic necessitated innovation, whether organizations were prepared or not. Evolution of resources and services is in line with adaptive capacity concepts which state that existing practices, resources, and behaviors will need to change as social and environment changes occur [5]. Extreme events, like a pandemic, have been shown to create pressure and drive quick evolutionary change [27]. Early on, questions emerged about how physicians and scientists could keep pace with the needs of a pandemic that was evolving daily. Informatics teams were required to expand their support at their institutions and across the CTSA consortium [3].

Demonstrating their ability to innovate and find pragmatic solutions, UC Davis CTSC collaborated in the development of StudyPages, a high-functioning participant recruitment and engagement platform for clinical research, assisting the Division of Pulmonary and Sleep Medicine with the recruitment, examination, and treatment of a diverse participant population into an industry-funded COVID-19 vaccine trial. Their StudyPages team portal had several unique features that allowed research teams to track, analyze, and report study participation over time. With 24,000 views of the study, 3500 people expressing interest via an online screening survey online, and 1281 “sign-ups” in a single day, this innovative and efficient approach culminated in a robust study population and dosing of the first 80 subjects within 2 weeks [28]. Making it easier for potential participants to find COVID-19 clinical trials to test experimental medicines, a special COVID-19 filter has been added to the University of Minnesota’s CTSI’s StudyFinder, a website that helps Minnesotans identify studies that need volunteers [29].

The Miami Clinical and Translational Science Institute (Miami CTSI) created a variety of initiatives in response to the pandemic, including those supported by informatics. Through NIH funding, the Miami CTSI collaborated with partners to create the Community-Engaged Research Alliance Against COVID-19 in Disproportionately Affected Communities (CEAL) initiative with a focus on: COVID-19 awareness and education among minority communities across the USA, especially among African Americans, Hispanics/Latinos, and American Indians; promotion and facilitation of inclusion and participation of these groups in vaccine and therapeutic clinical trials to prevent and treat the disease. Miami CTSI also utilized their clinical and translational research infrastructure to create a COVID-19 data dashboard and a COVID-19 Feasibility Committee [30].

In need of innovative solutions are many technical, policy, and organizational issues that constrain translationally relevant data sharing, “including, for example, data on drugs or other interventions intended for or currently being commercialized, data on drugs and devices that were approved by regulatory agencies, particularly those that are now off-patent, and data of various types from EHRs” [31]. To innovate on their adaptive and overall capacity, clinical and translational organizations could learn from and build on the following exemplars of academic and industry translational data transparency and sharing platforms and registries suggested by Austin: the NIH National Library of Medicine ClinicalTrials.gov database, Drugs@FDA and NCATS Inxight Drugs resources (https://drugs.ncats.io/), Open Data Portal of drug screening and animal model data (https://opendata.ncats.nih.gov/), N3C enclave for COVID-19 EHR data (https://covid.cd2h.org/), the Yale Open Data Access (YODA) Project (https://yoda.yale.edu), and the TransCelerate platform for clinical trial data (https://www.transceleratebiopharmainc.com/initiatives/historical-trial-data-sharing).

## Flexible Forward-Looking Decision-Making and Governance

An important aspect of adaptive capacity is anticipating change and planning for the future [5]. Developing efficient governance structures and a culture of shared vision is critical for informatics team’s capacity to adapt quickly. Institutions are the “rules, decision-making procedures, and programs that define social practices, assign roles to the participants in such practices, and govern the interactions among the occupants of those roles” [32]. Bookman et al. [3] offered their perspective on the state of research informatics in the midst of the ongoing pandemic. The researchers observed key features of informatics programs, their ability to quickly respond to the pandemic, and recommendations for the future. This information was supplemented by a survey of CTSA organizations to provide a more in-depth picture of the response to the pandemic from research informatics teams. The combined perspectives shared in the article provide insight for informatics ability to respond to emergent requirements and on the role of institutions and their decision makers. Seven pre-pandemic environments were described as contributing factors for the preparedness of CTSA informatics teams in responding to the pandemic as it unfolded: Governance, Tools, Common Data Models (CDM), Data terminology standards, Participant protections, Training, and Research use of the EHR. Within these concepts, several factors were identified that depend on flexible, forward-looking decision-making. These include allocation and sharing of resources, integration and adoption of tools, centralization and standardization of data, and the ability to be self-reliant through a culture of training.

Coller *et al*. [10] believe that innovation developed as a result of COVID-19 can lead to the adoption of new standards or “post-traumatic growth” that will make “the clinical research enterprise stronger, more resilient, and more effective.” An example of this is the Minnesota EHR Consortium, a statewide collaboration first of its kind in the USA, which pooled together patient data from the major healthcare providers in the state and began producing reports in less than a month from idea inception. This was made possible through pre-existing investments in clinical data infrastructure, as well as UMN CTSA hub’s strategic collaborations and inclusive leadership and decision-making [33]. According to the leader of the MN EHR Consortium COVID-19 Vaccine Project, Dr. Winkelman, “This important, intentional work to inform the equitable distribution of health care resources for Minnesotans is a true collaboration. …The ongoing partnership will not only guide decision-making processes related to COVID-19 vaccination distribution, but also provides the infrastructure needed for future public health crises.” The next capacity-building phase of the MN EHR Consortium is a CDC and Minnesota Department of Health funded infrastructure project that will develop the Observational Medical Outcomes Partnership (OMOP) common data model at 11 health systems across the state to more efficiently conduct disease surveillance, research, and quality improvement.

Planning for the future in the wake of the pandemic or other extreme event will require that informatics organizations remain flexible in governance structures and processes. Through the JCTS COVID-19 Special Edition Survey [23], CTSA hubs offered a range of responses to questions about procedures and decisions made during or after the pandemic. To deal with this emergency, hubs convened decision-making committees as quickly as possible, provided a separate policy-making body for human subjects research, and involved faculty and hubs more in decision-making regarding allowed research activities.

To prepare for the next emergency, hub representatives recommend organizational readiness to make decisions as quickly as possible, especially regarding risks/protections for the research and student environments, while keeping in mind that one size does not fit all with these environments. Effective communication was indicated as a key step for responding to future emergencies. We believe that there are key roles for informatics teams, tools, policies, and resources in the following hub-suggested practices in the emergency context:

- Developing a detailed plan in advance of any emergency and thinking ahead of time about ways to support research during times of "shutdown";
- Mobilizing key players for early collaboration;
- Developing a central location for key information;
- Communicating frequently and openly with the research community, addressing concerns, and providing guidelines and aid in adaptation of research; and
- Engaging the community much more quickly so they can become aware of clinical research and any research opportunities currently available.

## Implications and Conclusion

Significant implications for adaptive capacity and preparedness in informatics for CTSA program hubs were highlighted by the pandemic. Prior to the pandemic, CTSA hubs assets were defined by local and regional needs to access organizational electronic health records. However, through COVID-19, it quickly became clear that this local or regional focus was not sufficient and that a national level of hub coordination and cooperation was needed, particularly in the area of data availability and interoperability, as well as key infrastructure systems to support that.

CTSA informatics teams quickly moved to develop and support several significant local, regional, and national informatics and data innovations in response to the pandemic to speed the translation of discovery to improved human health. These efforts continue to highlight the value of the CTSA Program network to rapidly coordinate and harmonize resources and data across multiple systems. By its very nature, the pandemic challenged established processes and procedures at CTSA hubs and their informatics teams. The needs were increasingly greater than the established capacity could effectively handle. The pandemic’s sudden push for data and tools forced CTSAs to make decisions quicker and to remove unnecessary barriers. As demand increased, hubs began sharing information and best practices with greater frequency and quantity. Some of the challenges, lessons learned, and approaches to utilizing and advancing informatics – grounded in the pandemic experiences and captured by the Environmental Scan – are summarized in Table 1.

**Table 1.** Challenges for informatics in the context of emergency and approaches to address them (derived from the AC&P E-Scan)

Challenges for informatics in the context of emergency	Approaches for using and advancing informatics in the context of emergency
Lack of harmonized data in multiple databases.	Adapt existing resources for generating and managing information. Leverage and expand existing collaborations. Build centralized resources designed to aggregate/harmonize clinical data across organizations to enable rapid integration. (Asset Base)
Lack of access for the clinical and research community to a wealth of COVID-19 clinical data sitting in widely distributed databases.	Optimize collaboration in research networks. Pool resources and harmonize electronic health record data into a common data model across hubs, health systems, scientists, and other partners in different areas. (Institutions & Entitlements)
Increased demand for resources related to the use of cutting-edge informatics. Informatics knowledge and information are not systematically accessible by CTS stakeholders.	Cultivate a culture of Open Science and Data Sharing promoting FAIR (Findable, Accessible, Interoperable, Reusable) principles. Systematically capture informatics challenges and best practices to share via multiple channels. (Knowledge, Information, and Learning)
Engagement of a diverse participant population in pandemic-related and other research. Need for massive, interoperable clinical and research data during an emergency.	Utilize pragmatic information technology solutions that help engage and inform the community about research. Create collaborative, advanced analytics platforms and strategies to enable emergency-related data collection and novel analyses. (Innovation)
Limited availability and interoperability of clinical and research data, as well as a lack of effective communication and collaboration between key stakeholders within clinical and translational enterprise.	Integrate advanced informatics into translational research, community engagement, and workforce training. Prepare and adapt collaborative analytics approaches and platforms for future emergencies. (Flexible Forward-Looking Decision-Making)

COVID-19 has been a shared lived experience, no matter what the location. It is important to consider how we can learn from this experience. In the systematic review of all globally published 2,212 papers (from January to October 2020), describing new machine learning models for the diagnosis or prognosis of COVID-19 from CXR or CT images, Roberts *et al.* [34] found that “none of the models identified are of potential clinical use due to methodological flaws and/or underlying biases,” which is “a major weakness, given the urgency with which validated COVID-19 models are needed.” As translational science professionals and stakeholders, we must emphasize the importance of discussing and learning from such debacles, as well as ask ourselves: *What lessons from previous crises did we use in this one? What lessons from this crisis are we going to use in the next one?* As we supposedly live in the age of cutting-edge science, technology and information, questions like these must have been keeping many of us scientists awake at night.

Moving forward, CTSA hub informatics teams are expected to play a vital role in supporting public health regionally and nationally, although much work is required to codify the lessons learned from the pandemic into flexible practices. Investments must be made at all levels of the CTSA Program network to ensure that the consortium is prepared in an ongoing manner for large and small threats to the system. Tools, resources, practices, and policies should be evaluated so that responses to future crises can be streamlined and effective, while supporting ongoing efficient processes in day-to-day operations. Ongoing attention and support for an evolving informatics and data landscape, including open science, interoperability, and quality improvement initiatives are also required to support the increasingly critical role of informatics across the CTSA Program network.

## Select Examples and Resources

- Informatics Enterprise Committee – Understanding enterprise data warehouses to support clinical and translational research (2020)
  Qualitative study to assess the implementation of electronic data warehouses (EDW4R) for the purposes of research and how CTSA Hubs have operationalized EDW4R support.
- University of Minnesota – Sharing aggregated EHR data to improve Minnesota’s response to COVID-19 | Minnesota Electronic Health Record Consortium COVID-19 Project: Informing Pandemic Response Through Statewide Collaboration Using Observational Data
  Describes the creation of the Minnesota EHR COVID Consortium which was designed to pool together patient data from the major health care providers in the state. The Consortium went from idea to producing reports in less than a month.

- University of Nebraska Medical Center (UNMC) – A Framework for Bringing Secondary Analysis of EHR Data to Geographically Dispersed Clinician Scientists
  Poster presented at the 2020 ACTS Conference describing a potential framework for EHR systems to act as a biomedical repository for research.
- University Of North Carolina Chapel Hill – TraCS aids research mission of new UNC clinic to help patients with Post-COVID syndrome – https://clic-ctsa.org/news/tracs-aids-research-mission-new-unc-clinic-help-patients-post-covid-syndrome
  Description of North Carolina Translational and Clinical Sciences Institute’s effort to create a database collecting patient data once they leave the clinic in an attempt to better understand the impact on the patient experience during and after infection.

- Virginia Commonwealth University – VCU Wright Center informatics team fights COVID-19 with data – https://clic-ctsa.org/news/vcu-wright-center-informatics-team-fights-covid-19-data
  Provides a behind the scene description of how the VCU Wright Center informatics team supported data needs for nearly 50 pandemic related research projects.
- UCSF – Harvard Medical School – Five Questions with Griffin Weber – https://clic-ctsa.org/news/five-questions-griffin-weber
  Griffin Weber, co-faculty lead for Harvard Catalyst’s Informatics team, answers five questions about how his work supports COVID-19 research.

- View the AMIA Webinar Recording "Updates from the NIH National COVID Cohort Collaborative: An AMIA Public Policy Presentation" – https://clic-ctsa.org/news/view-amia-webinar-recording-updates-nih-national-covid-cohort-collaborative-amia-public-policy
  Link to AMIA webinar recording on the NIH National COVID Cohort Collaborative.
- University Of California Davis – Applying CTSA Principles to a COVID-19 Vaccine Trial – https://clic-ctsa.org/posters/applying-ctsa-principles-covid-19-vaccine-trial-2
  Poster presented at the 2020 fall virtual CTSA program meeting applying CTSA goals to a vaccine trial (includes audio).

- University Of Miami School Of Medicine – Miami Clinical and Translational Science Institute: Leading a collaborative effort to facilitate COVID-19 research and education for researchers and community stakeholders – https://clic-ctsa.org/posters/miami-clinical-and-translational-science-institute-leading-collaborative-effort-0Poster presented at the 2020 fall virtual CTSA program meeting describing the rapid response of Miami CTSI to develop key infrastructure supporting researchers and community stakeholders.
